# Growth rings of Brazil nut trees (*Bertholletia excelsa*) as a living record of historical human disturbance in Central Amazonia

**DOI:** 10.1371/journal.pone.0214128

**Published:** 2019-04-03

**Authors:** Victor L. Caetano Andrade, Bernardo M. Flores, Carolina Levis, Charles R. Clement, Patrick Roberts, Jochen Schöngart

**Affiliations:** 1 Instituto Nacional de Pesquisas da Amazônia, Manaus, AM, Brazil; 2 Department of Archaeology, Max Planck Institute for the Science of Human History, Jena, Thüringen, Germany; 3 Departamento de Biologia Vegetal, Instituto de Biologia, Universidade Estadual de Campinas, Campinas, SP, Brazil; 4 Forest Ecology and Forest Management Group, Wageningen University & Research, Wageningen, The Netherlands; Assam University, INDIA

## Abstract

The Brazil nut tree (*Bertholletia excelsa*) is an iconic and economically valuable species that dominates vast swathes of the Amazon Basin. This species seems to have been an important part of human subsistence strategies in the region from at least the Early Holocene, and its current distribution may be a legacy of past human settlement. Because *B*. *excelsa* is a long-lived pioneer tree it requires natural or human disturbances to increase light availability in the understory for a successful establishment. However, it remains unclear how the long-term population dynamics of this species have been shaped by pre-colonial and post-colonial human practices. Here, we use tree-ring analyses to look at changes in growing conditions over the past 400 years in a Brazil nut tree population in Central Amazonia. We identify changes in tree recruitment and growth rates associated not only with regional climatic variability, but also major political and socio-economic activities recorded by historical documents in the vicinity of Manaus. We demonstrate that the expansion of a post-colonial political center (Manaus) from the middle of the 18^th^ century onwards coincided with a reduction in recruitment of *B*. *excelsa*. We argue that this hiatus suggests the interruption of indigenous management practices, probably due to the collapse of pre-Columbian societies. A second recruitment pulse, and unprecedented cycles of growth release and suppression, aligns with a shift to modern exploitation of the forest into the 20^th^ century. Our findings shed light on how past histories of human-forest interactions can be revealed by the growth rings of trees in Amazonia. Future interdisciplinary analysis of these trees should enable more detailed investigation of how human forest management has changed in this part of the world, through pre-colonial, colonial, and industrial periods of human activity, with potential implications for conservation.

## Introduction

The Amazon Basin has provided a crucial window into the long-neglected extent of pre-Columbian human occupation and manipulation of tropical forest environments [1–7]. Forests of this region have often been argued to be ‘pristine’ or home to only small-scale human occupation and use prior to the arrival of European explorers in the 16^th^ century [8, 9]. However, recent archaeobotanical, archaeological, palaeoenvironmental, and ecological research has highlighted extensive and diverse evidence for plant domestication [3, 10], plant dispersal [3], forest management [11], and landscape alteration [1] by pre-Columbian societies. Nevertheless, while much attention has been focused on the cultivation of ‘crops’ in a traditional sense–including manioc, chili peppers, peanuts, squash, and maize [3, 7, 10]–trees have remained somewhat neglected in archaeological and historical studies of past human land management in the Neotropics. This is significant because many economically important trees dominate modern Amazonian forests, and some of these have undergone domestication processes [1, 12]. Therefore, understanding the changes in forest management witnessed by Amazonian forests over the course of the last four centuries has significant implications for ongoing human interaction with these threatened ecosystems.

The widespread Brazil nut tree (*Bertholletia excelsa* Humb. & Bonpl., Lecythidaceae) has become an increasingly important part of such discussions. Evidence suggests it has been used by humans since at least 11,000 years ago [13], and that its modern distribution is intimately linked to pre-Columbian human settlement patterns and demography reflected by *terra preta* soils and geoglyphs [3, 14]. The Brazil nut is a hyperdominant species that plays a major role in forest composition, structure, and biogeochemical cycling across Amazonian forests [12, 15]. Today, the Brazil nut is one of the main non-timber forest products from the region [16], whose seeds are collected by Amazonian communities, locally processed, and sold worldwide. Due to their economic and ecological importance across the Amazon Basin, these trees are also an important symbol of modern conservation efforts [16]. The Brazil nut tree is a long-lived, light-demanding pioneer [17–19], and its survival and canopy ascension are dependent on favorable light conditions [17, 18]. Its life cycle has therefore tended to be linked to natural disturbance events, such as tree-fall gaps, extreme droughts/floods, fires, and blowdowns [20–24]. However, a focus on natural agents often neglects the fact that humans have also been agents of Neotropical forest disturbance since the end of the Pleistocene [5, 25, 26].

Recently, dendroecological studies have emerged as a promising avenue for the investigation of disturbance patterns in tropical forests [18, 20–22, 24, 27–29]. Retrospective, multidisciplinary analyses of tropical tree rings can make important contributions to understandings of the ecology and longevity of tropical tree species [18, 30, 31], biomass productivity [32], forest dynamics [20, 21, 28], and the relationship between climatic fluctuations and tree growth [27, 33, 34]. Patterns of establishment and abrupt changes in tree growth provide insights into past local environmental conditions [20–22]. Events of growth releases and suppressions, which are based on the relative growth rate changes that trees experience during their lifetimes, are examples of disturbance indicators [21, 28, 35]. Stand structure and tree crown illumination profiles are analyzed in order to produce data on the current status of forest dynamics, these complementary methods associated with tree-ring analysis data provide the most comprehensive picture of historical stand development [20, 22]. While there has been an increasing interest in past human interactions with important tree products in the Amazon Basin, such as those of the Brazil nut [14], detailed studies of changes in human management of trees in the region have been limited. The management of this species often involves practices that include the clearance of the understory, opening of the forest canopy, liana cutting, and active protection of individuals [26], which makes the study of its rings promising for the detection of anthropogenic disturbances.

Given that the Brazil nut has been consistently linked to human subsistence and land-use practices, information on the local history of human management may be fundamental to understanding the dynamics of forests dominated by this species [36, 37]. Here, in an area of Central Amazonia with high Brazil nut density (locally known as *castanhais*), we compare tree-ring data from trees with available historical information on the political, economic, and human demographic changes in the region. We reconstruct the patterns of recruitment and growth of trees, and characterize the current status of the forest structure and tree crown illumination conditions. We gathered historical information about the Mura indigenous people, who inhabited the region before the establishment of the Portuguese colonial administration and witnessed their own population decline from the 18^th^ century onwards [38, 39], followed by the emergence of a new post-colonial society [40]. Our approach, based on interpretation of tree rings, provides a picture of the life histories of these nut trees and how they correlate with pre- and post-colonial human forest management. Understanding how forest management has changed following the arrival of European colonizers and the rise of industrial powers over the course of the past five centuries has implications for the future of sustainability and conservation in Amazonia [1, 5].

## Materials and methods

### Study area

**Fig 1:**
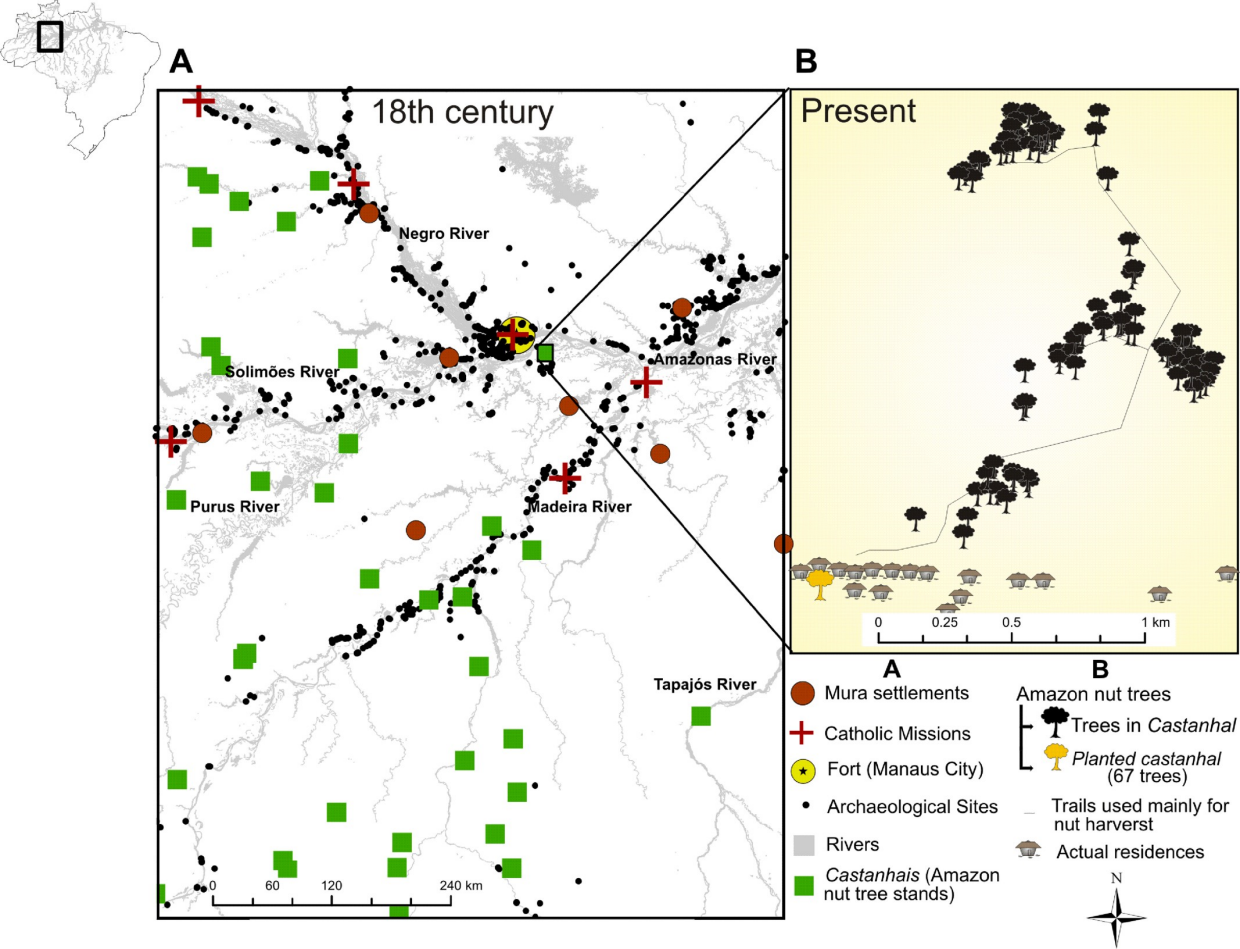
Past and present of the study area. (A) Overview of territorial occupation during the 18th century showing the major Mura settlements and Catholic missions, the Portuguese military base (today the city of Manaus), known archaeological sites of currently undefined ethnic relationship, and the *Bertholletia excelsa* populations. (B) Spatial distribution of Brazil nut trees in the *castanhal* near Purupuru Lake, including trails used by the local population and the 61 recently planted trees (represented by the yellow tree). Information in relation to Mura settlements, Catholic missions, *castanhais*, archaeological sites, and the river network come from Nimuendaju [39], Leite [43], RADAMBRASIL [44], AmazonArch (https://sites.google.com/view/amazonarch), and ANA/BRASIL (www.ana.gov.br), respectively.

Authorization to conduct this study was obtained prior to fieldwork through the Brazilian System of Protected Areas programme (SISBIO No 54510). We studied a Brazil nut stand, *castanhal*, located 30 km south of Manaus (Fig 1A), near Purupuru Lake (3°21'54.1" S, 59°51'27.5" W). The *castanhal* is located in a Central Amazonian forested landscape rich in other useful tree species, such as *Hevea brasiliensis* (Euphorbiaceae) and *Elaeis oleifera* (Arecaceae), popularly known as *seringueira* and *caiaué*, respectively [41]. The site has a seasonal precipitation regime with annual rainfall between 2000–2600 mm, and a distinct dry season from July to October [42]. The area is a low elevation plain, composed of small creeks (*igarapés*) and dikes connected to upland forests (*terra firme*) covering an area of about 30 ha [41]. The Brazil nut trees are aggregated in upland forest regions, with some individuals distributed along trails used today by the local population (Fig 1B). A village with a few families, whose subsistence is based mainly on farming and extractivism, is located less than one kilometer from the *castanhal*, where a single family planted 61 trees in an 8 ha area–we refer to this as the *planted castanhal* (Fig 1B). Local families report that the older trees of the *planted castanhal* were planted during the recent reoccupation of the area 70 years ago by their parents and grandparents. Tree-ring samples from these trees were not collected due to the particular significance of these individuals to these villagers.

### Historical background

The Purupuru Lake region has a dynamic pre- and post-colonial history that is well-documented archaeologically and historically. Archaeological survey has revealed a high density of *terra preta* archaeological sites in region, mostly dated to between 2,500 and 500 years BP [45]. *Terra preta* sites contain soils enriched with phosphorous, calcium, magnesium and other nutrients, and archaeological artefacts (such as pottery and bones), as an intentional or un-intentional result of human landscape management and long-term sedentary occupation [46, 47]. According to a wide range of historical sources, the region was occupied by the Mura indigenous peoples at the time of Portuguese arrival in 1657 [38–40, 48–51]. They practiced small-scale farming, mainly based on manioc and maize, as well as hunting, gathering, and fishing, with particular focus on the latter for obtaining protein rich resources [39]. The Mura built their houses in small groups, which were scattered far apart along the shores of a lake or river [39].

Historical sources, namely reports and letters of European settlers (i.e., priests, soldiers, scribes) [39], vary widely in their estimations of indigenous population size in the Purupuru Lake region in the 17^th^ century. Ethno-demographic estimates for Mura populations of the 17th, 18th, and 19th centuries range from 30,000 to 60,000, but are likely biased given their use by the colonial administration. Nevertheless, while exact numbers may not be available, there are clear periods in time when historical texts unanimously point towards periods of demographic transition. The late 17^th^ century and entirety of the 18^th^ century witnessed intense conflict between indigenous populations and the growing Portuguese colony [38–40, 48–53]. Initial conflicts represented resistance to arriving Catholic missionaries (1688–1750) and intensified during the Marquis of Pombal’s governance of Manaus city [40], causing the decline of local Mura populations as a result of disease, warfare, and relocation [42–45]. The 19^th^ and 20^th^ centuries were then marked by immigration fluxes of people associated with critical economic pulses [40, 52, 54, 55]. In particular, two Rubber Boom periods (1879–1912 and 1942–1945) [40, 55] witnessed the influx of thousands of people attracted by the extraction of a product with high global economic value [56].

### Tree sampling

We conducted this study at the beginning of the dry season (June/July) of 2016. All of the Brazil nut trees in the stand (N = 84) with diameter at breast height (DBH) > 10 cm were georeferenced and had their diameter measured. We collected cores from 78% of the trees (N = 67), so as to cover the largest possible range of diameters. We took two increment cores per tree at DBH using a 40 cm Haglöf increment borer (Långsele, Sweden) for the smaller trees (< 80 cm diameter) and an 80 cm drill made for a gasoline-drilling machine (Stihl BT45) for the larger trees (> 80 cm diameter). The bore-holes were later filled with carnauba wax (*Copernicia prunifera*, Arecaceae) to prevent pathogen damage. The cores were glued on wood supports and had their surfaces sanded with decreasing grit sizes (up to 600) to increase the distinctiveness of the growth rings, which are characterized by alternating fibrous zones and parenchyma tissue [18, 27]. We also georeferenced and measured the DBH of all trees in the *planted castanhal* (Fig 1B) (N = 61).

### Illumination profile

All individuals in the *castanhal* were categorized on a five-point scale illumination index of crown exposure (CE) levels [57]. Scores ranged from 1 to 5, where 1 is no direct lateral or overhead light, 2 is direct lateral light and no overhead light, 3 is partial overhead light, 4 is 90–100% of the crown area receiving direct overhead light, and 5 is full overhead and lateral direct light. CE values of all trees were estimated by the same observer. We are aware that estimates based on this scheme are only gross estimates [22], but they are also somewhat repeatable and accurate [58], and have been adapted and used in Neotropical forests [22, 59]. In any case, this provided an idea of the present competitive status of the trees in the *castanhal*. We related crown exposure levels (CE) with DBH using multinomial logistic regressions [22, 59] in SPSS 25 [22].

### Tree-ring analysis

Tree rings were identified on all cores using a 40x magnification stereomicroscope. Ring width was measured along the radii to the nearest 0.01 mm using a digital measurement table (Lintab, Rinntech, Germany), simultaneously producing curves of historical radial increments [18]. For each tree, ring-width measurements of different cores were visually cross-dated by using the TSAP-Win (Rinntech, Germany) program [18] and averaged. We corrected each increment value by multiplying it by a correction factor (difference between the summed diameter increments and actual tree DBH) and then transformed the radial increments to diameter increments by multiplying by two [28]. We estimated the number of rings in the missing part of the pith using the diameter obtained in the field and considering the pith position as a center. We chose this estimate considering that the majority of the samples were missing a few centimeters to reach the pith. By using the individual mean growth rate, we estimated the number of missing rings as less than 10 for 45% (N = 30) of the samples. Among the samples, 70% (N = 47) had their ages estimated as explained above, 16% (N = 11) presented entire cores, and 14% (N = 9) were discarded because the cores ended too far from the center of the tree.

Unlike other tree-ring studies that used entire stem discs of *B*. *excelsa* [5, 16], this study is based on the non-destructive sampling of cores, as harvesting is prohibited [60], which partially limits the capacity to analyze entire radii from the largest trees. Age estimation based on tree-ring analysis covered 69% (N = 58) of the trees. For the other portion (i.e., non-cored trees and those with a significant portion missing from the pith), we applied a non-linear regression model based on the cumulative individual curves (r^2^ = 0.72, p < 0.001) [18]. This fitted model has previously been applied to two other Brazil nut populations in Central Amazonia [18]. We did not correct tree ages for the time required to reach coring height (DBH). Juvenile individuals of *B*. *excelsa* can remain in the understory for a relatively long time before being released [18], which means that both average age and its variation could be estimated incorrectly. However, the probability of survival in the understory for this species is rather low; over a six-year period only two in ten trees survive [17]. We tested the occurrence of modality on our combined tree age profile by using the dip test [61], and the Gaussian finite mixture model to infer the number of clusters. We performed this analysis with the Mclust package [62], in R Software [63].

The individual diameter increment curves obtained by ring measurement were used to estimate the year-by-year growth change percentage (%GC), as suggested by Nowacki and Abrams [35]:

$$\%GC_{i}=\left\{\left(M_{2}‑M_{1}\right)/M_{1}\right\}\;x\;100$$

where *%GC*_*i*_ is the percentage of growth change for year *i*, *M*_*1*_ indicates the preceding 10-year mean diameter growth (including year *i*) and *M*_*2*_ is the subsequent 10-year mean diameter growth. This method expresses the growth change as a percentage of the previous growth period. Comparisons of sequential 10-year ring-width means were used to detect sustained growth increases or decreases that indicate changes in resource availability, while discounting strong inter-annual influences caused by climate variability [21, 35]. The first and last ten years of the individual tree-ring series were excluded from the analysis due to the definition of the equation [35]. Growth releases were defined as values above 100%GC and growth suppressions as values below -50%GC, both during at least five consecutive years [5, 35]. We also analyzed the median value of the year-by-year increment of each individual’s growth change percentage (%GC) to assess the changes in accelerated/retarded growth trends over time among all trees in the *castanhal*.

Finally, we qualitatively analyzed the occurrence of synchronous suppression events in relation to records of the main extreme maximum water levels at the port of Manaus since 1903 [64]. We used these records because the *castanhal* is located slightly downstream of Manaus, in a region of small creeks and oxbow lakes that receives its inflow from the Amazon River. We believe that the flood regime could have had a negative impact on the growth of Brazil nut trees, since the species is considered best adapted to *terra firme*. Local inhabitants reported that in years with very high water levels, the bases of trees can be covered by water, resulting in hypoxic or anoxic conditions that can negatively impact Brazil nut growth [23]. In addition to this, we also compared local and regional historical records of social, demographic, economic, and political events (see section on historical background) to patterns of tree growth in the *castanhal*.

## Results

### Brazil nut population structure and dynamics

**Fig 2:**
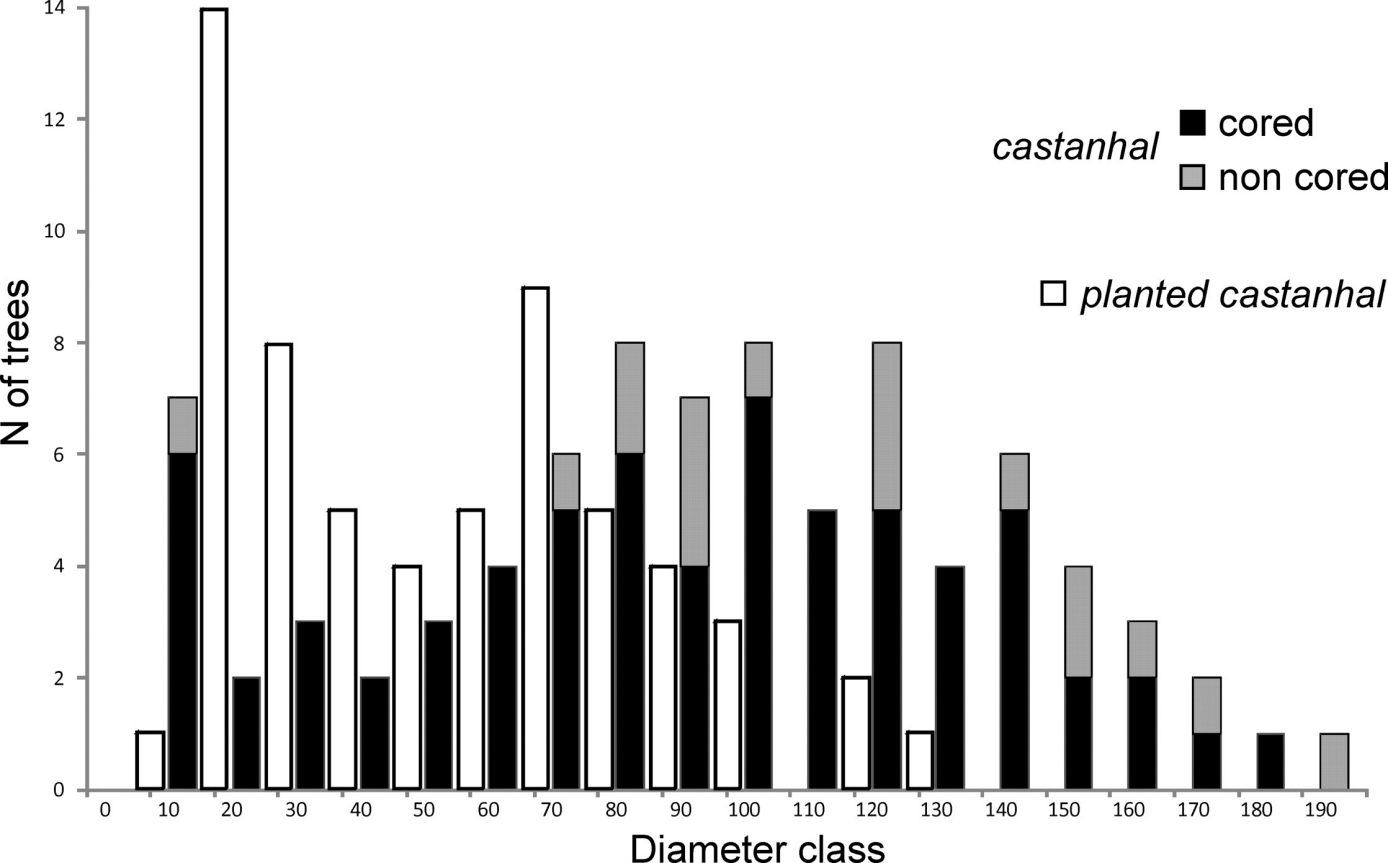
Frequency of Brazil nut trees by diameter class in the Purupuru Lake castanhal. Black bars represent the number of cored trees, gray bars represent the number of not cored trees, white bars represent trees in the *planted castanhal* that were not cored.

The *B*. *excelsa* population (*castanhal*) contains 84 individuals with a density of 3.2 ± 2.1 trees ha^-1^. Tree DBH measurements ranged from 10.0 to 192.6 cm, with a mean diameter of 97.8 ± 45.3 cm, and a mean basal area of 0.9 ± 0.7 m^2^. The sizes of all of the sampled trees (n = 84) approached a normal distribution (Lilliefors *p* = 0.2), with a majority in the intermediate diameter classes (DBHs 80–160 cm, 59.5%). The *planted castanhal* had a much higher density of 30.8 ± 9.2 trees ha^-1^, but trees have lower average basal area of 0.7 ± 0.4 m^2^ due to the lower mean diameter of 49.1 ± 29.7 cm. The non-normal distribution between diameter sizes of these trees (Lilliefors *p* = 0.016) confirms the information provided by the current residents that old trees are underrepresented in the *planted castanhal* (Fig 2).

The majority of the Brazil nut trees (92%, N = 77) had category 5 crown exposure (CE), which means that they receive full overhead and lateral direct light. Not all of these trees are emergent above the canopy; some are located beside trails that are constantly maintained during the annual harvest of nuts. CE levels increased with increasing DBH (S1 Fig), with a significant correlation (Nagelkerke r^2^ = 0.94). No trees were registered in category 1 (no direct lateral or overhead light) and 3 (overhead direct light < 90%) and only two trees are assigned to category 2.

**Fig 3:**
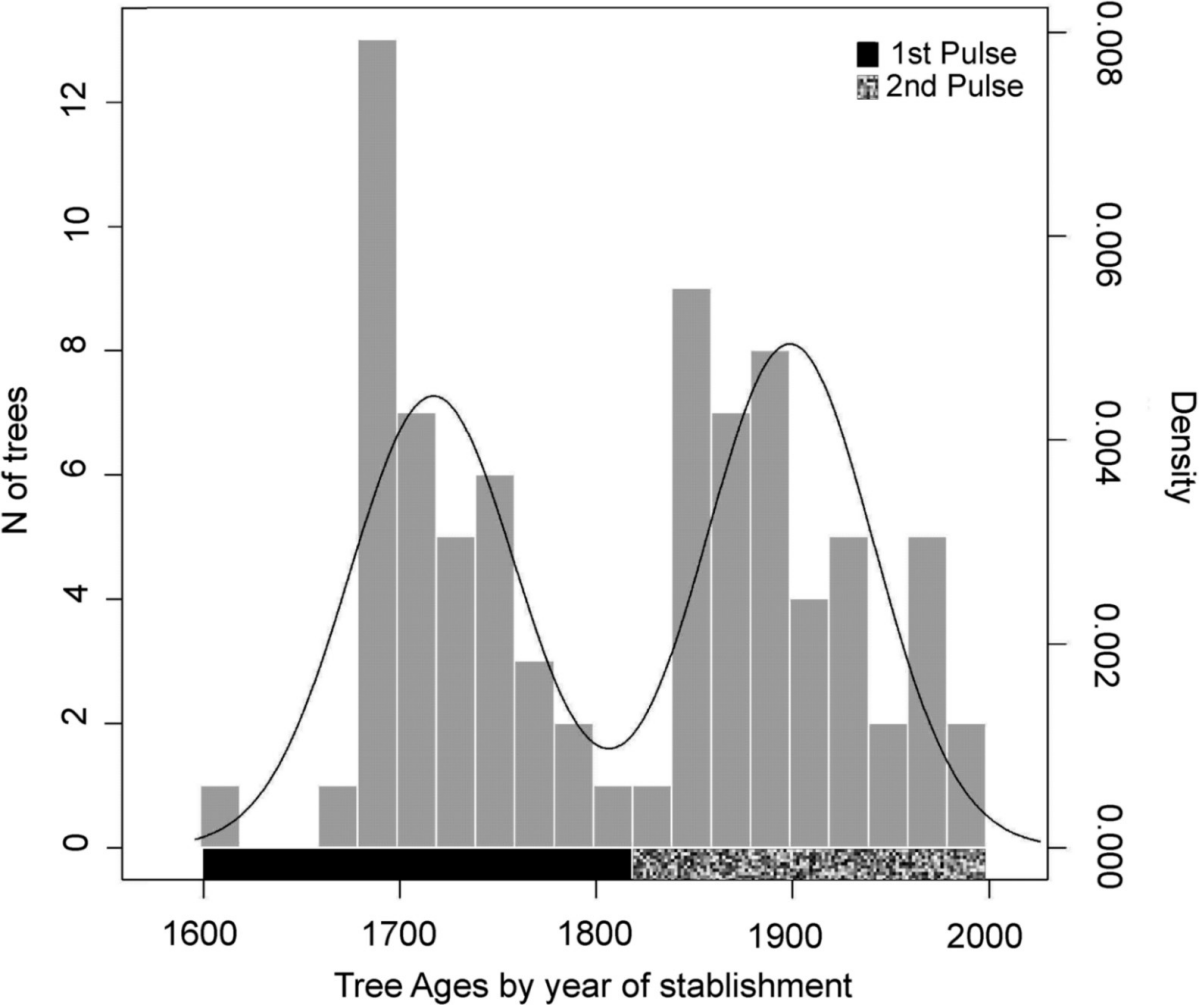
Recruitment histogram with estimated density curve of the Brazil nut stand at Purupuru Lake, Central Amazonia. The black line is a curve of probability density relative to the age distribution estimates based on bootstrap simulations, gray bars are the number of trees in 20-year intervals, the hatched and black bars represent the two clusters we call recruitment pulses.

**Fig 4:**
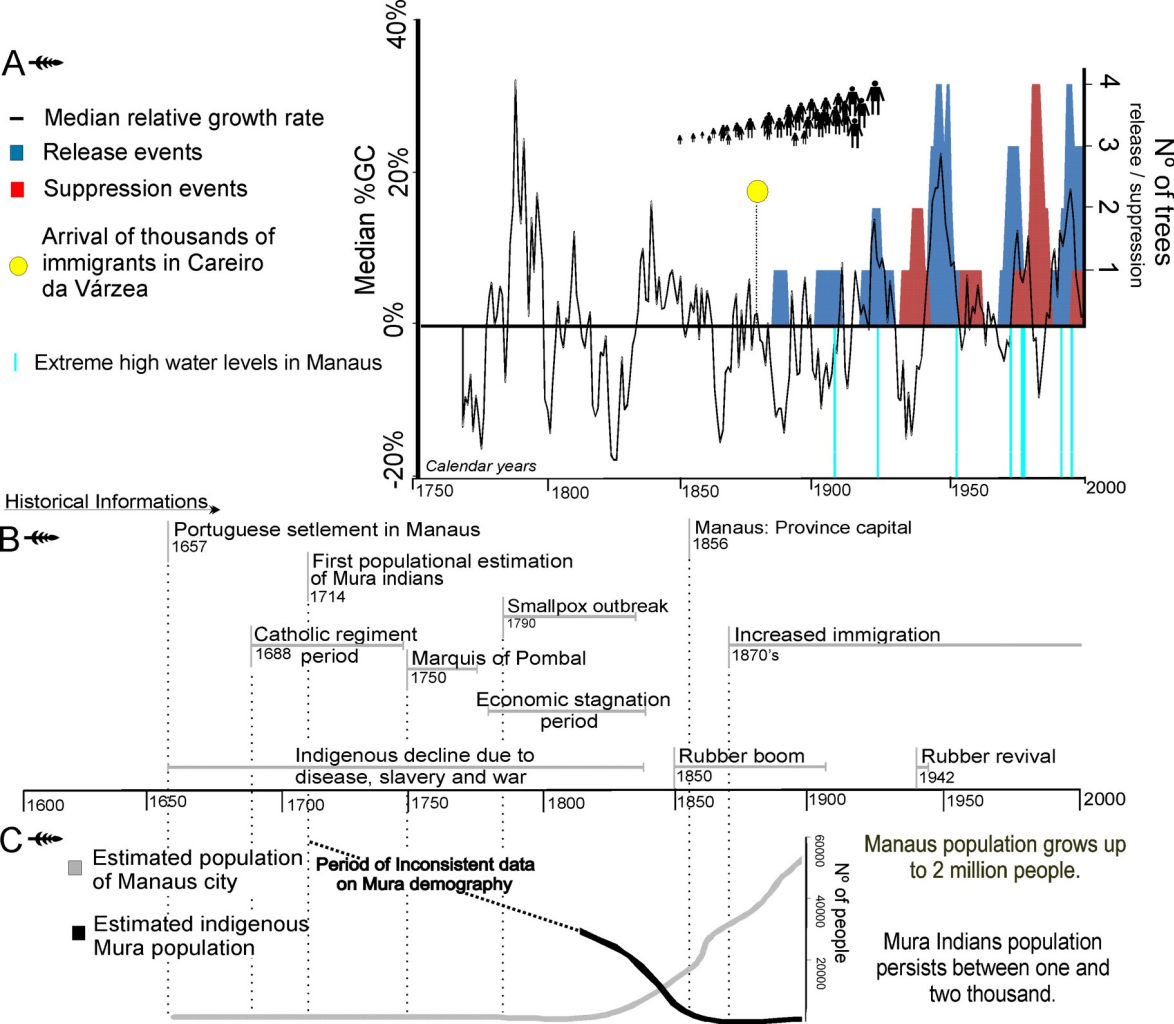
Integration of historical, climatic and forest dynamics information. (A) Black line represents the median of percent growth change (%GC) among cored trees in the *castanhal* population. Red bars indicate the number of trees passing through suppression events and blue bars indicate the number of trees passing through release events. The human icons represent population growth in the study area stimulated by migration. The yellow dot indicates when hundreds of families were registered in the municipality to work in rubber extraction [55]. Extreme high water levels (1909, 1922, 1953, 1971, 1975, 1976, 1989, 1994, 1999) are based on records of water levels at Manaus since 1903 [64]. (B) Events of historical importance to understand the territorial dynamics of the study region in Central Amazonia. (C) Estimated population for indigenous Mura and colonizers (references are cited in the text).

By combining the results of the age estimation model and tree ring analysis, the age structure of the *castanhal* was divided into two distinct recruitment pulses (Fig 3), as the dip test indicated significant bimodality for age distribution (D = 0.07, P < 0.001). This was confirmed by the Gaussian finite mixture model that fitted the age distribution in a two-cluster, non-parametric, univariate and with equal variances model. The component encompassing the older trees is termed the 1^st^ recruitment pulse and has 41 individuals with ages ranging from 198 to 412 years, while the component with the younger trees is termed the 2^nd^ recruitment pulse with 43 trees with ages varying from 27 to 176 years (Fig 3). We estimate that the oldest living tree in the *castanhal* was recruited in the early 17^th^ century, before continuous European presence in the area. The recruitment in the first pulse continued for two centuries (Table 1), although recruitment slowed as European presence and influence became stronger (Fig 3, Fig 4B). The second pulse started at about the same time as the Rubber boom.

**Table 1:** Description of the Brazil nut tree recruitment pulses at Purupuru Lake, Central Amazonia. Period of establishment based on our combined estimate of tree ages, mean diameter of trees (± standard deviation), and mean age in each pulse based on the Gaussian finite mixture model (± bootstrap confidence interval).

Recruitment Pulse	Period of establishment	Nº of trees	Average diameter (cm)	Average age (yr)
**1st Pulse**	1604–1818	41	132.7 (±29.3)	303 (289–314)
**2nd Pulse**	1840–1989	43	64.7 (±33.2)	119 (106–134)

Analysis of the median percent growth change (%GC) showed periods of accelerated growth in the decades 1780–90, 1830–40, 1920, 1940, 1970, and 1990 (Fig 4A). The longest period of median %GC > 0 was 18 consecutive years during the 1830-40s, which followed the longest period of median %GC < 0, which was 17 consecutive years during the 1810-20s. The other long periods of decelerated growth occurred in the decades of 1770, 1860, 1880, 1900, and 1930. The analysis of individual %GC revealed that 17% (N = 14) of the trees underwent at least one release event during their growth trajectory, and 8% (N = 7) went through at least one suppression event. These release/suppression events began to occur in our analysis after 1886 (Fig 4A). Peaks in the number of trees experiencing simultaneous release events (four) occurred around 1950 and 1998, and a single peak in suppression events (also four) occurred around 1985. Other samples showed no significant changes in growth trajectories. Suppression events did not show any clear correlation with extreme maximum water levels in Manaus [64] (Fig 4A).

### Comparison with historical data

Information about local human history is fundamental in order to understand both the present structure of the Brazil nut population and its temporal dynamics. This information includes dated events of territorial occupation, migration flows, and resource exploitation, which are all expected to influence forest management whether intentional or not. In 1657 the first Portuguese expedition to the region occurred [40] (Fig 4B), and a decade later the construction of the fort of *São José do Rio Negro* initiated colonization of the site on which the city of Manaus is located today [40] (Fig 1A). This settlement soon became a nucleus of indigenous slave trading and a source of "Indian manpower" for the Portuguese crown [40]. The Mura people, who occupied an extensive area on the other side of the river where the *castanhal* is located, resisted Portuguese colonization with extreme effectiveness [38, 48–50]. Indeed, the initial opposition of the Mura was effective to the point that the first Portuguese settlers of the Manuas locality avoided crossing the Amazon River or leaving the limits of their settlements [40].

In the late 17^th^ century, Franciscan, Carmelite, and Jesuit missionaries arrived at *São José do Rio Negro* (Fig 4B) and the settlement expanded, uniting indigenous peoples of different ethnicities [40]. During this period, colonizers (i.e., missionaries, soldiers, colonists) made constant incursions into the forest to search for indigenous slaves and forest products [40, 52]. Until 1750, the Portuguese crown granted land and power to church entities [52, 53], while the Mura people remained in their forest territory, not appearing in lists of “pacified Indians” [40]. Attacks between settlers and the Mura were frequent during this period [38, 48–50]. In 1750, the Marquis of Pombal became the main political figure in Portugal and started a new developmental project for Amazonia (Fig 4B). His objective was to create a wage-labor reserve with indigenous peoples and colonists [52]. Portuguese military forces increasingly came into conflict with indigenous people who disobeyed government rules. Mura populations were attacked every year, and those captured in their villages were enslaved or killed [39]. By 1774, the colonists demanded extermination of the Mura and other recalcitrant Indians [39], resulting in the decimation of indigenous populations [65] and the emptying of the forest [66].

In the early 19th century, Brazilian Amazonia was experiencing a stagnant economy and smallpox outbreaks [52, 53] (Fig 4B), and human population density in the vicinity of Lake Purupuru was low. From the mid-19^th^ century, with the expansion of demand for rubber, Amazonia experienced accelerated economic growth [40, 52]. The commercial exploitation of Brazil nut seeds occurred in parallel with rubber extraction, and export flows increased for both products [54]. In 1848, the Brazil nut began to appear in export records [54]. Efforts by the Brazilian government to meet the demand for labor in Amazonia brought thousands of migrants, especially from the northeast of Brazil, and the forest began to be reoccupied [54, 55, 67] (Fig 4C). These new colonists brought their own management strategies and different tools than those used by indigenous peoples. Hundreds of rubber trees in our study area retain characteristic signs of extraction by tappers from this period [41].

After the economic stagnation caused by the collapse of the rubber boom in 1920, the international demand for rubber increased again from 1941 to 1945 [40], leading to the departure of 72,278 people from the urban center of Manaus to nearby cities and the interior of the state in search of this product [68]. During World War II, the Brazilian rubber economy experienced an unexpected strong increase in exports due to the international control of the product by Japan and the growing demand for military equipment. This put Amazonia back on the export map, in a period that we term the rubber revival (Fig 4B). During this period, the village where our study area is located expanded with the arrival of these new rubber tappers [55]. These people encouraged the emergence of Brazil nut trees in their backyards, the *planted castanhal* (Fig 1B). Currently, Purupuru Lake has a large number of inhabitants on its banks, mostly settlers from these 20th century migrations who are involved mainly in small-scale farming, Brazil nut extraction, hunting, and the exploitation of other forest products.

## Discussion

Based on our analyses of more than three centuries of Amazon nut tree population dynamics, we suggest that this emblematic and highly useful tree species has been intimately connected with humans through forest management practices. We observe that fluctuations in tree recruitment coincide with periods of human occupation, and that a hiatus in tree establishment suggests the impact of political changes that led indigenous populations to abandon the forest and their management practices. The beginning of the first recruitment pulse in the early 17^th^ century reflects the average maximum age of Brazil nut trees (*c*. 360–440 years) reported in Amazonia [18, 28, 69]. Information on the longevity of *Bertholletia* is still controversial in literature [18], and it is possible that older dates will emerge in future [70], though our findings fit with a significant amount of existing research. It is interesting to note that the beginning of the first recruitment pulse is roughly contemporary with a significant increase in the arrival of European colonizers into the region, which may suggest that colonists had some influence on the recruitment of the older living trees. However historical records point to separation between the European colonists and the *castanhal* area at that time [40], and it is more plausible that these older trees represent the average natural life-cycle of Brazil nut trees in the region. For the Mura peoples, who extensively occupied the region in the past, collecting fruits in the forest was an important secondary activity in their economy [39], and they used Brazil nut as a food resource [51]. The Mura peoples abandoned the region in the middle of the 18^th^ century during the climax of the war between Portuguese colonizers and Mura populations, when most of those who survived surrendered to government forces [38, 48]. During this period, human population density was low, disease outbreaks impacted the region, and the Purupuru Lake site was a location vulnerable to attacks by both indigenous inhabitants and colonizers [40] (Fig 4B, Fig 4C and Fig 1A). As a consequence of this abandonment, dense forest cover probably formed, hindering Brazil nut tree recruitment [71].

It therefore appears that the decreased tree recruitment in the middle 18^th^ century can be attributed to the declining Mura population [38, 39], and the abandonment of their forest management. The decline in Brazil nut recruitment from 1760–1840, followed by a new recruitment pulse, is an indication that disturbance regimes changed at this point in time. This change could be related both to extreme climatic events, such as fires and windstorms [20, 24], or forest management [72]. We argue that one of the dominant triggers of tree recruitment in the studied *castanhal* is, in fact, human action. Practices that provide benefits for useful tree species, such as non-useful plant removal (e.g., creating forest gaps and cutting lianas) and fire management results in understory clearance and periodic canopy openings [26], which are fundamental factors for the recruitment of the sun-loving Brazil nut tree [17]. Fire management has also been commonly associated with *terra preta* formation elsewhere across the Amazon Basin and is known as being key part of long-term agroforestry strategies [4, 7, 26, 41, 47, 73, 74].

Periodic windstorms, extreme droughts, and fire are major causes behind large-scale and moderate intensity disturbances in tropical forests [20, 21, 24]. Fire has been commonly used to manage vegetation in Amazonia since pre-Columbian times [26], and charcoal is abundant in forest soils of this region [41]. Brazil nut trees have high regeneration capacity after fire disturbances, provided that high light conditions are maintained [17], which makes it perfectly adapted to fallow agricultural practices [75, 76]. Severe windstorms that occur at decadal or centennial scales [20, 77], and impact several hectares almost instantly [78, 79], are a possible alternative explanation for the recruitment increase around 1820. However, it is difficult to identify the occurrence of such a disturbance based on the analysis of a single species, especially given that this species is so closely linked to human activities. To identify large-scale disturbances in the study area it would be necessary to analyze trees of different species to identify similar patterns for recruitment and growth trends [20, 24] and build robust, local palaeoenvironmental datasets [80].

The period from 1860 to 1910 is characterized by high Brazil nut tree recruitment associated with mostly negative growth changes in the *Bertholletia* population, something that is difficult to explain. A recently published reconstruction of the precipitation based on tree rings from *Cedrela odorata* (Meliaceae) in Eastern Amazonia [81] suggests prolonged dryness from around 1864 to 1881, with seven consecutive severe drought years (1864–1871). The correlation maps between the tree-ring chronology of *Cedrela* and the precipitation regime includes Central Amazonia where our study site is located. In this period, we found growth changes of the *Bertholletia* population to be consistently negative and it is possible that consecutive severe drought conditions caused a persistent decrease in diameter growth. In support of this idea, two multi-annual throughfall exclusion (TFE) experiments in old-growth Amazonian rainforests (terra firme) at Caxiuanã and Tapajós National Forest Reserves showed a 20–30% reduced leaf area index, increased mortality rates, especially of large trees, and a reduction of gross primary production, which increases the vulnerability of these ecosystems to fire [82]. The canopy opening caused by increased mortality and declining leaf area index, however, possibly enhanced solar radiation at the forest floor and favored the recruitment of Brazil nut trees [17].

However, even if such natural disturbances occurred, the effects that they had on the forest were probably enhanced by human influences as the second tree recruitment pulse coincides with the beginning of the arrival of immigrants coming mainly from the northeast of Brazil to work in rubber extraction [55]. Abrupt changes in tree growth patterns are normally the result of inter-tree competition and light availability, which sustain these changes over years [21, 28]. Growth releases began to occur during the first rubber boom in the late 19^th^ century, in the exact decade when hundreds of families, or a few thousand immigrants, were registered in the municipality of Careiro da Várzea, where the *castanhal* is located [55] (Fig 4). Due to the fact that Brazil nut exploitation occurs during the rainy season, coinciding with the period in which there was no extraction of rubber, the greater the number of rubber tappers, the greater the exploitation of Brazil nut expected, as this was the main secondary extractive activity [54]. Changes in tree growth could also be related with extreme floods that occurred in the region, especially because locals reported that the water can cover the base of the trees in extreme flood years. Although extreme floods have significant impacts on tree growth [23, 83–85], we found no clear evidence for such natural disturbance agents as triggers for synchronous events of release and suppression in our study (Fig 4A).

Disturbances in the 20^th^ century were somewhat different from those of the previous periods, causing up to four synchronous releases and suppressions (Fig 4). The absence of releases and suppressions in our dataset during the indigenous period does not indicate absence of forest disturbance; such events could have occurred for fewer trees with old records or because disturbances were more subtle across the area. Due to the fact that rubber trees are seldom abundant in the forest, rubber tappers frequently protect existing seedlings and saplings of rubber trees, plant seeds and seedlings, and clear the understory vegetation surrounding rubber trees according to practices described by Levis et al. [26]. During both rubber booms these practices were occasionally reported and rubber stands (*seringais*) were created in some areas [86]. Due to the importance of this product, and the commercial pressure placed upon it, rubber tappers are likely to have created more intense forest disturbance than indigenous societies. We suggest that the 20^th^ century disturbances may have been influenced by the intensive global commercial pressure during the second rubber boom, associated with the increasing demand for this product in World War II [55]. This demand led the Brazilian inhabitants of the study area to create the *planted castanhal* in the 1940s, further emphasizing the importance of this species in the local community.

The influence of human management on present forest structure is remarkable. Brazil nut density in the old *castanhal* (3.2 trees ha^-1^) is almost ten times lower compared to the *planted castanhal* (30.8 trees ha^-1^), and the diameter distributions reveal that recruitment of Brazil nut trees was enhanced in the *planted castanhal*, while it simultaneously declined in the old *castanhal* (Fig 2). The decline of Brazil nut tree recruitment after the second pulse in the old *castanhal* is probably a result of less frequent human visits to this area. Yet, it should also be noted, that only trees with diameter above 10 cm were sampled in this study and smaller recruiting trees may not have been recorded. In the old *castanhal*, we observed that regeneration was common along trails that connect the smaller aggregates of Brazil nut trees (Fig 1B), a pattern also reported for forests occupied by the Kayapó people of southeastern Amazonia [87]. Light conditions below the canopy of the old *castanhal* might be unfavorable for recruitment, as almost all individuals > 10 cm DBH are established in the canopy receiving direct overhead light (S1 Fig). Variations in canopy opening patterns between different Brazil nut stands are probably influenced by intensity of disturbances caused by humans [72]. Activities such as maintenance of trails and nut harvesting are likely to create more forest disturbance and, therefore, better light regimes that promote Brazil nut growth. The oldest trees in the *planted castanhal* (N = 7) were 72 years old and had a mean diameter of 92 cm, which implies a mean diameter increment of 12.8 mm yr ^-1^, similar to growth rates of commercially planted Brazil nut trees near Manaus [18]. Moreover, the estimated mean diameter increment rates and tree ages documented in this study are comparable with others using tree-ring analysis [18, 28], repeated diameter measurements [59], dendrometers [88], and radiocarbon dating [69], which support the hypothesis that Brazil nut distribution across the basin was partly driven by past human activity [14, 89].

## Conclusions

The potential of living trees to reveal insights into past human-environment dynamics reinforces the interpretation of Amazonian forests as sites not only of ecological heritage, but also of cultural significance [5]. This has major implications for how we view the modern conservation of these ecosystems, and highlights the importance of long-term datasets when considering appropriate strategies for future management and conservation. Our study also emphasizes the importance of taking into account historical information and human-mediated disturbance histories when considering the evolution and dynamics of modern forests. In Central Amazonia, human management of tropical forests has undergone a number of drastic changes in the Late Holocene, with the arrival of European colonists and, later, with global industrialized societies. These changes seem to have left their mark on Brazil nut tree structure and dynamics in the Purupuru Lake region. We suggest that the study of living trees has much to offer ecologists, archaeologists, and conservation specialists alike in the context of discerning past human management practices and their influences on forest dynamics. The combination of dendrochronology with other biochemical techniques, including radiocarbon dating, stable isotope analyses, and genetics, has significant potential to provide more highly resolved into these human-mediated changes that have left a lasting legacy in modern forests. Our findings suggest that recruitment of Brazil nut trees is associated with the actions of both pre- or post-colonial human societies The hiatus in Brazil nut tree recruitment coincides with the collapse of indigenous societies throughout Central Amazonia and the loss of their management practices, followed by modern patterns of forest exploitation. The study of Brazil nut stands, and other economically significant species, across Amazonia has the potential to reveal just how widespread these changes in forest management were, as well as their lasting legacy in terrestrial ecosystems that are rapidly vanishing from the face of the planet.

## Supporting information

S1 Fig Exposure of tree crowns to illumination. Multinomial logistic regression between diameter of Brazil nut trees (N = 84) and Crown Exposure (CE) classes (1–5) in the Purupuru Lake *castanhal*, Nagelkerke R^2^ is 0.94. CE class 1 is no direct lateral or overhead light, 2 is direct lateral light, no overhead light, 3 is partial overhead light, 4 is 90–100% of the crown area receives overhead direct light, and 5 is full overhead and lateral direct light.
(TIF)
